# Proteomic and Biological Analysis of the Effects of Metformin Senomorphics on the Mesenchymal Stromal Cells

**DOI:** 10.3389/fbioe.2021.730813

**Published:** 2021-10-05

**Authors:** Mustafa Burak Acar, Şerife Ayaz-Güner, Zeynep Gunaydin, Musa Karakukcu, Gianfranco Peluso, Giovanni Di Bernardo, Servet Özcan, Umberto Galderisi

**Affiliations:** ^1^ Genome and Stem Cell Center (GENKÖK) Erciyes University, Kayseri, Turkey; ^2^ Department of Molecular Biology and Genetics, Faculty of Life and Natural Science, Abdullah Gül University, Kayseri, Turkey; ^3^ Institute of Health Sciences, Erciyes University, Kayseri, Turkey; ^4^ Erciyes Pediatric Stem Cell Transplantation Center, Department of Pediatric Hematology and Oncology, Faculty of Medicine, Erciyes University, Kayseri, Turkey; ^5^ Research Institute on Ecosystems (IRET), CNR, Naples, Italy; ^6^ Department of Experimental Medicine, Luigi Vanvitelli Campania University, Naples, Italy; ^7^ Department of Biology, Faculty of Science, Erciyes University, Kayseri, Turkey; ^8^ Center for Biotechnology, Sbarro Institute for Cancer Research and Molecular Medicine, Temple University, Philadelphia, PA, United States

**Keywords:** mesenchymal stem cells, senescence, senolytics, senomorphics, aging

## Abstract

Senotherapeutics are new drugs that can modulate senescence phenomena within tissues and reduce the onset of age-related pathologies. Senotherapeutics are divided into senolytics and senomorphics. The senolytics selectively kill senescent cells, while the senomorphics delay or block the onset of senescence. Metformin has been used to treat diabetes for several decades. Recently, it has been proposed that metformin may have anti-aging properties as it prevents DNA damage and inflammation. We evaluated the senomorphic effect of 6 weeks of therapeutic metformin treatment on the biology of human adipose mesenchymal stromal cells (MSCs). The study was combined with a proteome analysis of changes occurring in MSCs’ intracellular and secretome protein composition in order to identify molecular pathways associated with the observed biological phenomena. The metformin reduced the replicative senescence and cell death phenomena associated with prolonged *in vitro* cultivation. The continuous metformin supplementation delayed and/or reduced the impairment of MSC functions as evidenced by the presence of three specific pathways in metformin-treated samples: 1) the alpha-adrenergic signaling, which contributes to regulation of MSCs physiological secretory activity, 2) the signaling pathway associated with MSCs detoxification activity, and 3) the aspartate degradation pathway for optimal energy production. The senomorphic function of metformin seemed related to its reactive oxygen species (ROS) scavenging activity. In metformin-treated samples, the CEBPA, TP53 and USF1 transcription factors appeared to be involved in the regulation of several factors (SOD1, SOD2, CAT, GLRX, GSTP1) blocking ROS.

## Introduction

Following genotoxic stress, caused by either external or internal stimuli, cells undergo senescence, which arrests cell division and induces a loss of cell functions (Campisi and d’Adda di Fagagna, 2007). In terms of evolutionary history, senescence arose as mechanism to counteract cancer since it blocks proliferation of cells with damaged DNA. Nevertheless, the accumulation of senescent cells within tissues and organs contributes to organismal aging and, paradoxically, may promote the onset of cancer (van Deursen 2014). These events mainly occur due to the paracrine activity of senescent cells, which secrete a plethora of proteins and other macromolecules that are collectively known as Senescence Associated Secretory Phenotype (SASP). The SASP induces the senescence of neighboring healthy cells, promotes inflammation phenomena, remodels the tissue’s extracellular matrix by causing the loss of tissue architecture, and sustains cancer growth through the activity of growth and survival factors (Ozcan, Alessio et al., 2016).

Some pioneering studies have shown that ablation of senescent cells in tissues prolonged health spans and reduced the risk of age-related pathologies (ARD) in a mouse model (Kim and Kim 2019). This finding paved the way for the development of a new class of drugs called senotherapeutics, which can modulate senescence phenomena within tissues and reduce the onset of ARD (Niedernhofer and Robbins 2018; Kim and Kim 2019; Myrianthopoulos et al., 2019). Senotherapeutics fall under two classes: senolytics and senomorphics. The senolytics selectively kill senescent cells by triggering apoptosis by blocking the survival networks. The senomorphics may act in one of two ways: they can revert the phenotype of senescent cells back to healthy cells or they can delay or block the onset of senescence following genotoxic stress (Kim and Kim 2019).

Metformin is a biguanide moiety drug that has been used for the treatment of type 2 diabetes for several decades. The metformin lowers the circulating glucose levels in patients with type 2 diabetes mainly by inhibiting hepatic gluconeogenesis (LaMoia and Shulman, 2021). Beyond these effects, metformin may exhibit anti-aging properties by preventing DNA damage and inflammation. Specifically, it prevents macromolecule damage by decreasing reactive oxygen species (ROS) synthesis *via* reverse electron flux and by inhibiting superoxide production through mTOR pathways (Valencia, Palacio et al., 2017). Given these phenomena, several clinical trials aimed at evaluating the effect of metformin on aging and ARD in non-diabetic patients have been conducted. For example, the trial named MILES (Metformin in Longevity Study) proposes a pilot investigation to examine the effect of metformin treatment on the biology of aging (ClinicalTrials.gov Identifier: NCT02432287). Another study is a double blind, placebo-controlled trial to evaluate anti-aging effects in adults with prediabetes (NCT03309007).

In spite of clinical trials on the possible benefits of metformin for ARD treatment, the mechanisms underlying these benefits are still not fully understood. In this context, the possible anti-aging effects of metformin on stem cells has not been investigated in detail. The onset of senescence in stem cell compartments has a great impact on health since stem cells promote tissue renewal and organismal homeostasis. Specifically, the mesenchymal stromal cells (MSCs) present in the stroma of several tissues contain a stem cell subpopulation that can differentiate into mesodermal derivatives (bone, cartilage, fat, etc.) and secrete dozens of factors that modulate functions of tissues’ immune systems and renewal processes (Squillaro et al., 2016). Given the key role of MSCs in the biological functions of the human body, their senescence can greatly impair health outcomes. It is thus important to evaluate senotherapeutics that can address MSCs senescence.

Several studies evaluated the effects of metformin on MSCs biology. Metformin treatment protects MSCs from DNA damage events, delays senescence, reduces their level of reactive oxygen species, and promotes differentiation phenomena (Gu et al., 2017; Kuang et al., 2020; Kim et al., 2021). Additionally, metformin may enhance the immunomodulatory potential of MSCs (Park et al., 2019). Other studies have addressed the negative effects of metformin on MSCs. This drug may reduce cell survival and trigger apoptosis (He et al., 2019). These contrasting results may be due to many factors, including: 1) metformin concentration and duration of treatment, 2) the type of MSC (from adipose tissue or bone marrow), and 3) species under investigation (human or mouse).

Indeed, the methodology of several studies involved treating cells with a micromolar concentration of metformin for a few days. This approach is far from current clinical protocols, which consider long-term treatment at higher concentrations. evaluating the effect on MSCs of prolonged metformin treatment in the range of therapeutic concentration at the millimolar level (Hess et al., 2018) is worthy of study.

We then evaluated the effect of metformin on the biology of human adipose MSCs treated with therapeutic doses of metformin for 6 weeks. These treatments were in the 1–10 mM range according to findings evaluating serum metformin levels in patients (Hess et al., 2018). Our study was combined with proteome analysis of changes occurring in MSCs’ intracellular and secretome protein composition in order to identify molecular pathways associated with the observed biological phenomena.

## Results

### Metformin Delays Replicative Senescence and Protects From Apoptosis

**FIGURE 1:**
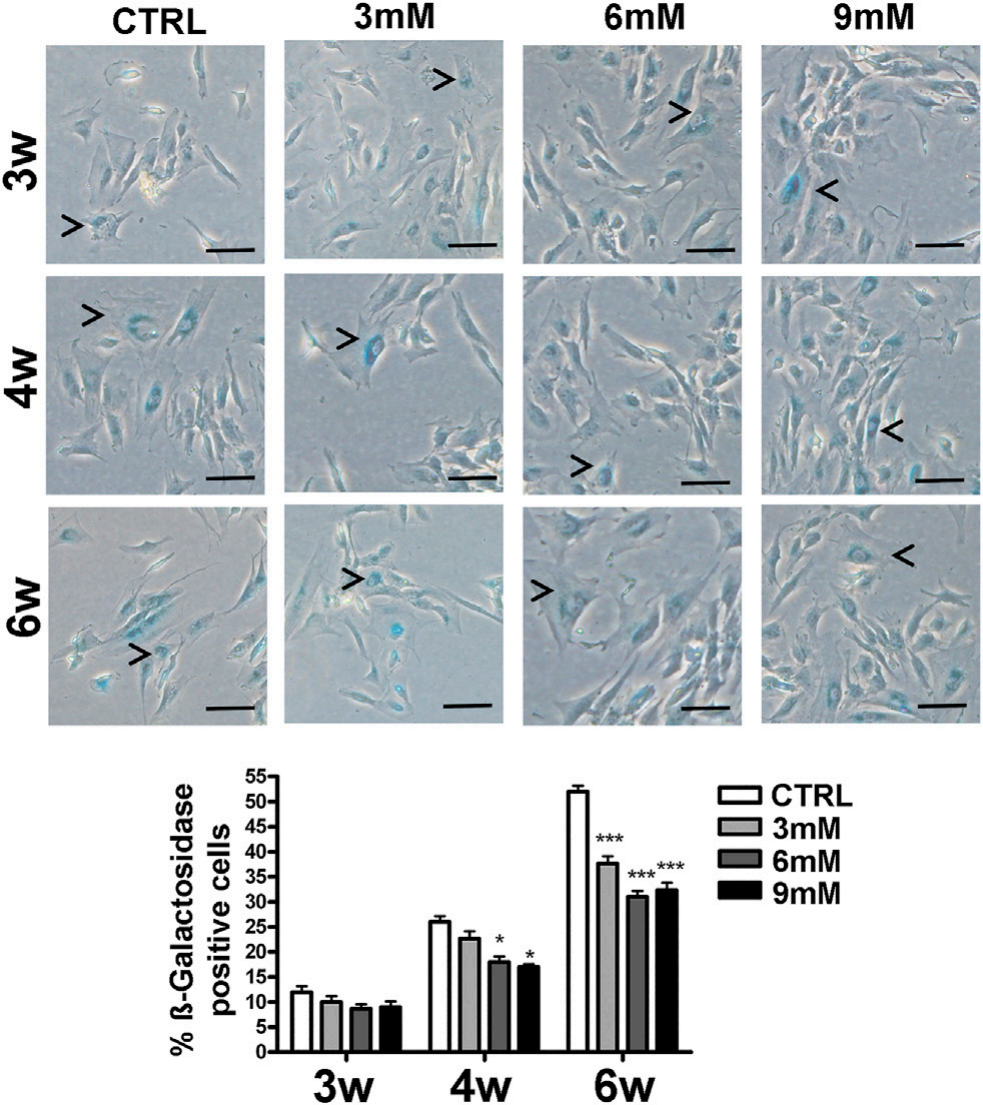
Senescence levels in MSCs cultures. This histogram shows the percentage of senescent cells after three (3w), four (4w) and six (6w) weeks of *in vitro* culture, either in the absence (CTRL) or presence of metformin. Data are shown with a standard deviation (SD) *n* = 3 *p < 0.05, ***p < 0.001. For each time point, the symbol (*) indicates the statistical difference between the control culture and those treated with metformin. The pictures show representative images of senescent cells that tested positive for the beta-galactosidase activity (blue). The arrow heads indicate some typical senescent cells, which show flattened morphology and blue staining in the perinuclear area. The bar corresponds to 100 microns.

**FIGURE 2:**
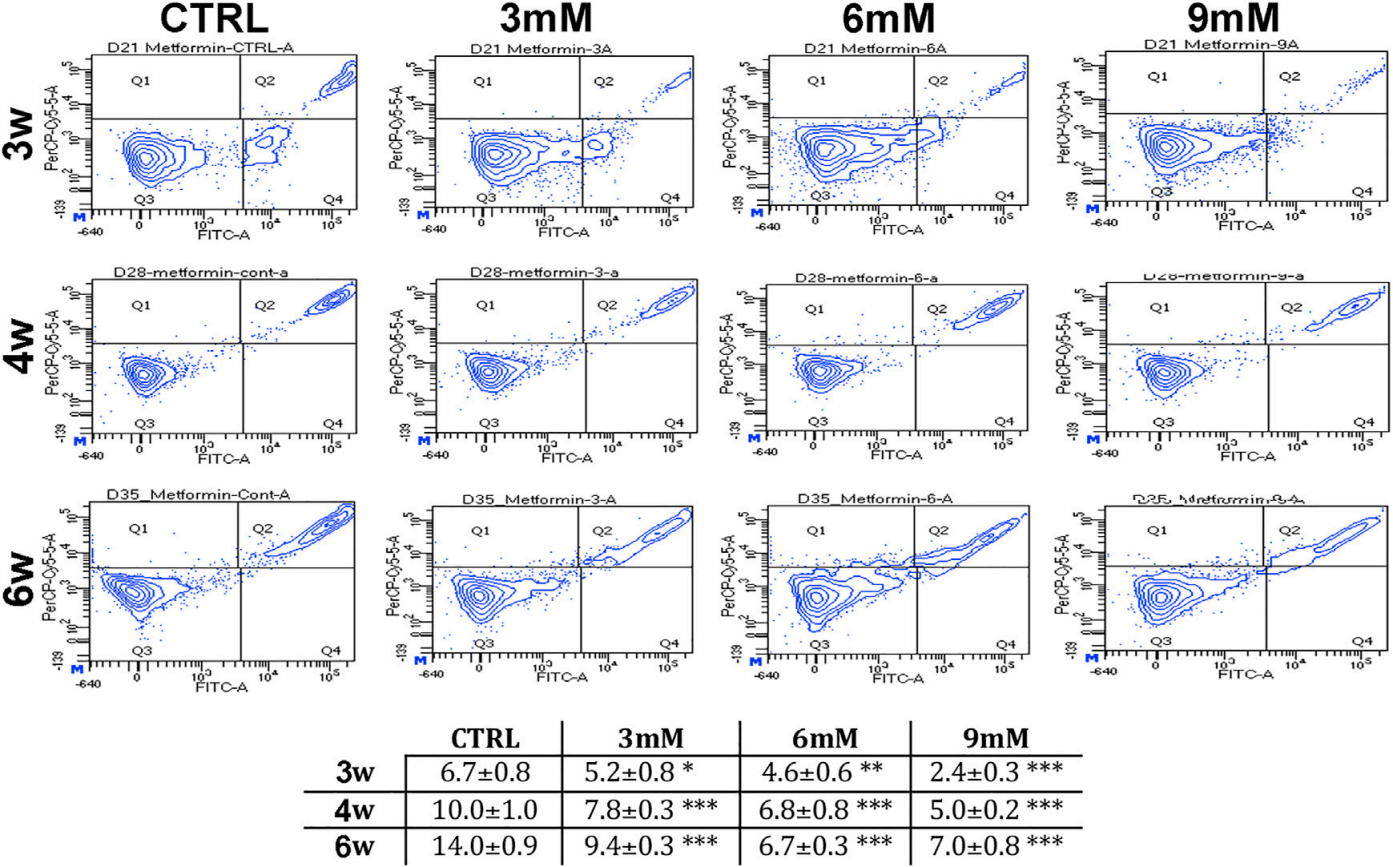
Apoptosis levels in MSCs cultures. This table shows the percentage of apoptotic cells after three, four and 6 weeks of *in vitro* culture, either in the absence (CTRL) or presence of metformin. Data are shown with standard deviation (SD) *n* = 3 *p < 0.05, **p < 0.01, ***p < 0.001. For each time point, the symbol (*) indicates the statistical difference between the control culture and those treated with metformin. The pictures show representative images of annexin-V detection by flow cytometry analysis. Apoptotic and non-apoptotic cells were identified by two separate dyes (Annexin V and 7AAD, respectively). The phosphatidylserine is bound by Annexin V (FITC-A labeled) on the external membrane of apoptotic cells, while 7AAD (PerCP-Cy5-5-A labeled) permeates and stains DNA in late-stage apoptotic and dead cells. Coloration enables the identification of three cell populations: non-apoptotic cells (Annexin V- and 7AAD+); early apoptotic cells (annexin V+ and 7AAD-); and late apoptotic or dead cells (Annexin V+ and 7AAD+). In our experimental conditions, both the early apoptotic and late apoptotic cells were grouped.

We incubated adipose-derived MSCs (replicative passage 3) with 3 mM, 6 and 9 mM of metformin and evaluated some biological parameters (cell cycle, apoptosis and senescence) at 3 weeks, 4 weeks and 6 weeks post-treatment. Control cultures showed a progressive decrease of dividing cells since the percentage of S-phase doubling dropped from 7.7% at 3 weeks to 2.1% at 6 weeks (Table 1). At two and 4 weeks of incubation with metformin, no significant differences between the control and treated samples was observed. However, at 6 weeks a higher percentage of cells in the S-phase was seen in treated samples. Specifically, MSCs incubated with 9 mM metformin showed 6.7% of cells in the S-phase (Table 1). The *in vitro* cultivation induces replicative senescence through telomer attrition. The percentage of senescent MSCs sharply increased from 11% at 2 weeks to more than 50% at 6 weeks. At this data-collection point, the metformin treatment had greatly reduced the percentage of senescent cells. In detail, incubation with 6 and 9 mM metformin almost halved the percentage of senescent cells (Figure 1). Prolonged cell cultivation leads to cell stress that, in addition to the onset of senescence phenomena, may trigger apoptosis. Indeed, after 6 weeks of cultivation, MSCs showed a significant growth of apoptotic cells (6.7% at 3 weeks vs. 14% at 6 weeks) (Figure 2). Of great interest, the metformin treatment also reduced the apoptosis level at all the analyzed time points (Figure 2).

**TABLE 1:** Cell cycle analysis.

	Cell cycle phase		
	G1/G0	S	G2/M
3 WK CTRL	69.9%	7.7%	17.8%
4 WK CTRL	81.1%*	4.1%*	11.6%
6 WK CTRL	87.6%**	2.1%**	9.5%**
3 WK 3 mM Met	66.9%	9.1%	19.7%
4 WK 3 mM Met	79.8%	5.2%	12.5%
6 WK 3 mM Met	77.5%	**4.5%#**	16.1%
3 WK 6 mM Met	68.1%	9.1%	19.2%
4 WK 6 mM Met	82.0%	4.7%	10.9%
6 WK 6 mM Met	76.9%	**4.7%#**	16.2%#
3 WK 9 mM Met	64.0%	8.9%	21.2%
4 WK 9 mM Met	81.4%	4.5%	11.8%
6 WK 9 mM Met	76.9%	**6.7%##**	14.6%

This table shows the percentage of cells in each of the different phase of cell cycles after 3, 4 and 6 weeks of *in vitro* cultures, either in the absence (CTRL) or presence of metformin. For each condition (CTRL, 3 mM, 6 mM and 9 mM metformin), the symbol (*) indicates the statistical difference between 3 weeks of treatment (chosen as reference) and the other time periods. For each time point (3, 4 and 6 weeks), the symbol (#) indicates the difference between the CTRL and the other conditions. The symbols * or # and ** or ## correspond to *p* < 0.05 and *p* < 0.01, respectively. The values in bold indicate the most significant changes in S phase following metformin treatment.

### Proteome Analysis of Cell Lysates and Secretomes of MSCs

**FIGURE 3:**
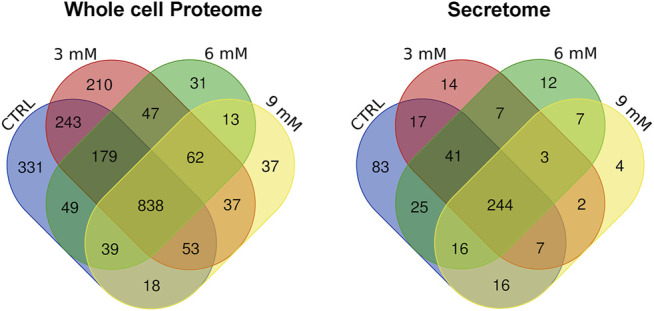
Venn analysis of proteins found in cell lysates and secretomes. These pictures show the proteins that occur in all the experimental conditions (CTRL, 3, 6, and 9 mM metformin treatment), and those that are only present in some.

Our study demonstrated that 6 weeks’ treatment with metformin produced the most striking changes in the analyzed biological functions; thus, we performed a LC-MS/MS analysis of whole cell proteome and secretome of MSCs cultivated *in vitro* for 6 weeks with or without metformin supplementation. For each experimental point we consider only proteins that were present both in biological and technical replicates. In control MSCs, the LC-MS/MS analyses of peptides identified 1,749 proteins in whole cell lysates and 448 proteins in the secretome. Similar numbers of proteins were identified in the metformin-treated samples (Supplementary Material S1). The Venn analysis of proteome demonstrated that in addition to a common core of proteins being present in all cell lysates or in all secretomes, each experimental condition exhibited specific proteins (Figure 3, Supplementary Material S2). This result indicates that metformin profoundly modified the cellular protein composition of MSCs as well as their secretomes. Next, we conducted several bioinformatics investigations to gain insights into the differences in protein composition between the control and metformin-treated MSCs.

We parsed the proteome profiles of our samples by Gene Ontology (GO) in order to determine the relative frequency of ontological terms associated with specific cellular functions. We accomplished this by performing analysis of the terms grouped in the GO biological process database. For each experimental condition, we identified one hundred ontologies and then used Venn-diagram analysis to combine the GO data and identify the biological processes that were affected by metformin (Supplementary Material S2). Interestingly, we found enrichments in metabolic processes—such as glycolysis, carbohydrate metabolic process, carboxylic acid catabolic process, and nucleotide biosynthetic process—in the whole-cell proteome of metformin-treated samples. These enriched ontologies were also identified in secretome samples of metformin-treated MSCs (Supplementary Material S2). This result is in keeping with the role of metformin in modifying cellular metabolism.

### Analysis of Canonical Pathways Shows That Metformin can Restore Signaling Associated With Normal MSC Functions

GO analysis found the enriched ontological terms in our samples but could not identify the most important proteins in several of the experimental conditions. In this context, we performed a canonical pathway analysis to identify the set of interactions for each protein in our datasets. This investigation allowed us to determine the most representative pathways that could have a functional impact on the observed biological phenomena.

For every experimental condition, the proteins present in cell lysate and the secretome could be attributed to hundreds of canonical pathways (Supplementary Material S3A, B). We fixed a high statistical significance (*p* < 0.001) cutoff in order to determine the pathways that were overrepresented in our samples. Also, with this limitation, we identified many pathways for each analyzed sample. The Venn diagram was then used to combine the data concerning all of the experimental conditions to find the specific canonical pathways of the control samples and of those treated with metformin (Supplementary Material S3A). We further limited our analysis by comparing the pathways exclusively present in the control or in metformin-treated samples, which were grouped together irrespective of drug concentration. This strategy allowed us to identify a few pathways that could be further investigated (Table 2). The control MSCs showed ten exclusive pathways, while the metformin-treated cells exhibited four specific canonical pathways. The majority (eight out of ten) of control pathways could be associated with the typical features of senescent cells. Senescent cells are resistant to death due to active anti-apoptotic signaling. They may lose their original functions, but they will remain highly metabolically active and synthesize dozens of factors that are released in the SASP (van Deursen, 2014; Kim and Kim, 2019; Myrianthopoulos et al., 2019). The control MSCs presented a nucleotide-excision repair pathway, a cysteine biosynthesis pathway and an iron homeostasis signaling pathway, all of which can be related to survival strategies used to cope with genotoxic stress events (Nakamura et al., 2019; Daher et al., 2020). These cells employed pathways associated with aerobic and anaerobic glucose catabolism (PFKFB4 signaling, Acetyl-CoA biosynthesis) and nucleotide metabolism (Ribonucleotide biosynthesis, pentose phosphate pathways, etc.). Additionally, they presented an active dopamine receptor signaling that could be responsible for impaired MSC functions. Indeed, dopamine signaling can block the wound healing activity of MSCs (Shome et al., 2012).

**TABLE 2:** IPA analysis followed by Venn diagram.

CTRL whole cell proteome	Metformin whole cell proteome	CTRL secretome	Metformin secretome
**Canonical Pathways**			
Dopamine Receptor Signaling	Aspartate Degradation II	Intrinsic Prothrombin Activation Pathway	Role of PKR in Interferon Induction
Cysteine Biosynthesis	Œ±-Adrenergic Signaling	Virus Entry *via* Endocytic Pathways	
Pentose Phosphate Path	Xenobiotic Metabolism PXR Signaling Pathway	Telomere Extension by telomerase	
Acetyl-CoA Biosynthesis	Gap Junction Signaling	Sertoli Cell-Sertoli Cell Junction Signaling	
PFKFB4 Signaling Pathway		Semaphorin Neuronal Repulsive Signaling	
Iron homeostasis signaling		Coronavirus Replication	
Ribonucleotide Biosynthesis		Agrin Interactions at Neuromuscular Junction	
NER (Nucleotide Excision Repair)			
Ethanol Degradation II			
Role of MAPK Signaling in Promoting the Pathogenesis of Influenza			
**Upstream transcription factors**			
HOXD3, FLI1, MEF2D, ETV4, IRF2, MYOD1	YY1, TP53, CEBPA, USF1	LMO2, LEF1, HSF1, LDB1, KLF4	FOSL1

Treatment with metformin profoundly modified the intracellular signaling in MSCs. We identified four specific pathways, and three of them were related to the recovery of cellular functions and a reduced presence of senescence phenomena. Alpha-adrenergic signaling is important for the physiological secretory activity of MSCs (Tyurin-Kuzmin et al., 2016). The xenobiotic metabolism PXR signaling pathway is associated with the detoxification activity that MSCs can exert on the surrounding environment (Oladimeji and Chen 2018). Furthermore, the aspartate degradation pathway is related to optimal energy production, which is based on the transfer of cytosolic NADH into the mitochondrial matrix (Munoz et al., 2014).

Also, we found specific pathways in the secretome of control MSCs that can be associated with senescence and SASP activity. Indeed, the telomeres extension through the telomerase pathway is associated with replicative senescence as it is well known that telomeres’ erosion occurs after prolonged *in vitro* cultivation (Victorelli and Passos, 2017). The virus entry via endocytic pathway is indicative of active the endo/exocytosis events that are responsible for SASP production and paracrine signaling among senescent cells and the surrounding healthy cells (Ayaz-Guner et al., 2020). The intrinsic prothrombin activation pathway could be associated with the pro-inflammatory activity of SASP (Chu, 2010). In the secretome of metformin-treated MSCs, we found only one specific pathway: the role of PKR (protein kinase R) in Interferon Induction and Antiviral Response. There are findings showing that MSC immunosuppressive properties are based on the activation of indoleamine-2,3-dioxygenase-1 through interferon-beta and PKR (Opitz et al., 2009).

### Putative Transcription Factors Regulating the Identified Canonical Pathways

Following the identification of significant pathways in our experimental conditions, we performed a regulatory network analysis by determining key transcription factors that are likely to be responsible for the changes observed in our data (Supplementary Material S4). We conducted an IPA upstream regulatory investigation followed by Venn diagram analysis to determine the transcription factors exclusively present in the control samples or the metformin-treated cultures (Supplementary Material S4, Table 2). We found that six transcription factors were exclusively present in the whole cell proteome and five transcription factors were only identified in the secretome of control cultures, respectively. In the metformin-treated cells, we found four and one transcription factor(s), respectively (Table 2). Most of the identified transcription factors have pleiotropic activities since they regulate many biological functions including antagonistic tasks, such as cell survival and apoptosis, depending on environmental factors. In this context, it is not a straightforward matter of attributing them a role in our experimental conditions. We tried to circumvent this difficulty by examining the proteins they putatively regulated in our samples (Supplementary Material S5). For example, in metformin-treated cells the TP53 transcription factor putatively regulates 381 proteins; while in the control sample, the HOXD3 factor regulates eight proteins (Supplementary Material S5). We focused our attention on proteins that were regulated by at least two of the identified transcription factors. This allowed us to identify some key proteins and their associated transcription factors in our datasets (see Tables 3 and 4).

**TABLE 3:** Proteins in the control datasets and the related upstream transcription factors. CTRL whole cell lysate.

Transcription factors	Total	Regulated proteins
FLI1 HOXD3	1	COL1A1
FLI1 MEF2D	2	COL1A2
		CCN2
ETV4 MEF2D	2	FN1
		VIM
ETV4 MYOD1	1	ACTA2
IRF2 MYOD1	1	EIF2AK2
**CTRL secretome**		
HSF1 KLF4 LEF1	2	FN1
		VIM
LDB1 LMO2	11	ACTB
		GSN
		SERPINE2
		MFGE8
		EFEMP2
		MYH10
		LGALS1
		ANXA1
		COL18A1
		SERPINF1
		FBLN1
HSF1 LEF1	6	MMP2
		ECM1
		CCDC80
		CTTN
		DKK1
		PENK

**TABLE 4:** Proteins in the metformin-treated datasets and the related upstream transcription factors. Metfomin whole cell lysate.

Transcription factors	Total	Regulated proteins
CEBPA TP53 YY1	3	PCNA
		COL1A1
		HSPA5
CEBPA TP53 USF1	3	SERPINE1
		FASN
		AKAP12
TP53 YY1	9	VIM
		DNAJB4
		RBBP4
		SFPQ
		TGM2
		MCM6
		ACTA2
		CRYAB
		TIMP1
CEBPA TP53	14	VCL
		SERPINB2
		ANXA1
		SOD2
		SOD1
		ACLY
		GAPDH
		THBS1
		AKR1B1
		PGD
		ASNS
		COL1A2
		GSTP1
		GLRX
TP53 USF1	4	P4HA1
		CAT
		MYH9
		BAX
CEBPA YY1	2	PFN2
		QKI
**Metformin secretome**		
FOSL1	7	MAP1B
		MMP1
		MMP2
		SERPINE1
		SERPINE2
		SPARC
		THBS1

In control cultures of MSCs, FLI1, MEF2D and MYOD1 in cooperation with other factors regulate the expression of proteins involved in cytoskeletal and extracellular matrix formation (e.g., Actin, Collagen, Fibronectin) (Table 3). All these proteins play a key role in senescence and its associated extracellular remodeling. They are listed in the senescence gene database (https://senequest.net). Also in the control secretome, LEF1 and LMO2, together with other factors, regulate many proteins that are part of cytoskeletal and extracellular structures. In the samples besides the control secretome, these transcription factors regulate the expression of all proteases that are involved in senescence (Serpins, Metalloproteases) (Table 3). All the listed proteins play a role in senescence as indicated in Senequest database.

In metformin treated samples, CEBPA, TP53 and YY1 regulate the expression of the genes that may be negatively associated with senescence (COL1A1; HSPA5) (Bigot et al., 2012; Li et al., 2014). There were other senescence-associated proteins whose expression is regulated by the identified upstream factors, such as Serpins and Timp1 (Table 4). Nevertheless, we observed many factors (SOD1, SOD2, CAT, GLRX, GSTP1) that neutralize ROS and hence prevent the onset of senescence (Davalli et al., 2016). Additionally, there were proteins (DNAJB4, CRYAB) involved in the regulation of protein folding even after stressful stimuli, such as those inducing apoptosis or senescence, impact the cell (Miao et al., 2019; Zhang et al., 2019) (Table 4).

## Discussion

The senomorphic drugs may represent a new tool for treating aging and its related pathologies. Many compounds for accomplishing this goal are under investigation; nevertheless, even if they are proved effective in *in vitro* and animal studies, their medical use is still hotly debated since senomorphics must be utilized for long periods of time in order to enact their anti-senescence effect. In this context, the metformin may represent a valid alternative since it is a drug with quite tolerable side effects even after years of treatment.

There are two classes of senescence: acute and chronic senescence. The first is due to acute genotoxic stimuli, such as exposure to chemical and physical agents that induce DNA damage. Chronic senescence may be triggered by cellular stresses that last for extended times, such as continuous proliferation and DNA replication. These events cause accumulated DNA damage through the arrest of cell proliferation and the impairment of physiological functions (replicative senescence). A senomorphic drug that has to be utilized in a long-lasting therapeutic regimen should play a major role in reducing and preventing the onset of chronic senescence.

In this context, for a complete assessment of metformin senomorphic properties, we evaluated its effects on MSCs, which play a key role in bodily homeostasis and tissue renewal. There are several findings that demonstrate the consequences of metformin treatment on MSCs biology. In a mouse model of kidney disease, a short pulse of metformin inhibited the acute senescence of bone marrow MSCs (Kim et al., 2021). Others have shown that metformin reduces the level of ROS and the onset of senescence in mouse adipose-derived MSCs (Marycz et al., 2016). Metformin may also play a role in the immunomodulatory potential of MSCs and in their differentiation properties (Gao et al., 2008; Gu et al., 2017). In addition to these positive effects, metformin may promote the apoptosis of MSCs and decrease their angiogenic capacity (Montazersaheb et al., 2018; He et al., 2019). The present study endeavored to combine this piecemeal information in a more comprehensive way. First of all, we focused our attention on the effects of metformin on MSCs replicative senescence since this drug should have senomorphic activity. We then combined biological data with the bioinformatical analysis of the MSCs proteome in order to identify key regulatory pathways and networks that could be associated with the observed biological phenomena.

The metformin treatment in the range of the therapeutic concentration reduced the replicative senescence of MSCs as evidenced by the decreased levels of beta-galactosidase activity and the presence of cells actively synthesizing DNA even after 6 weeks of *in vitro* cultivation. It is worth noting that we did not observe an increase of apoptosis following metformin treatment; rather, we detected a significant reduction of cell death associated with prolonged *in vitro* cultivation. This result contrasts data showing that metformin-induced apoptosis of MSCs when cells were incubated in media with a low glucose concentration. We performed our investigation on adipose-MSCs while others investigated cell death in bone marrow umbilical cord MSCs. This difference may partially explain this conflicting data, but further studies are needed.

The bioinformatics analysis of this proteome content allowed us to associated pathways and signaling networks with the observed biological phenomena. The cellular proteome and the secretome of MSCs cultivated *in vitro* for 6 weeks were enriched in proteins and pathways associated with senescence. This data further supports the presence of senescence phenomena as detected with biological assays. The continuous metformin supplementation during *in vitro* cultivation delayed and/or reduced the impairment of MSC functions as evidenced by the presence of specific pathways in the metformin treated samples. These pathways are 1) the alpha-adrenergic signaling, which contributes to regulation of MSCs physiological secretory activity, 2) the signaling pathway associated with MSCs detoxification activity, and 3) the aspartate degradation pathway for optimal energy production.

Of great interest, the senomorphics’ function of metformin seemed to be related to its ROS scavenging activity. In metformin-treated samples, the CEBPA, TP53 and USF1 transcription factors appeared to be involved in the regulation of several factors (SOD1, SOD2, CAT, GLRX, GSTP1) implicated in blocking ROS.

## Conclusion

Metformin prevents and/or delays the replicative senescence of MSCs. This result further supports the idea of using metformin as a means of senomorphics. The identification of key networks and proteins that are associated with metformin senomorphics functions suggests that this task was mainly accomplished through anti-ROS activity. This study paves the way for a more detailed *in vivo* analysis of metformin senomorphics effect on MSCs, since an effective anti-aging drug must preserve stem cells’ functions. Indeed, the available results from *in vivo* studies on animal models and the current metformin-based clinical trial for treatment of aging related diseases did not address the effect of such a treatment on MSC biology.

## Materials and Methods

### Culture of MSCs

Adipose tissue-derived MSCs were obtained from the American Type Culture Collection (ATCC PCS-500-011) and were grown in DMEM containing 10% FBS, 4 mM L-glutamine, 100 U/mL penicillin-streptomycin, and 5 ng/ml bFGF. Cells were provided at replicative passage 3 (P3) and were cultivated for up to 6 weeks either in presence or absence of metformin. All the biological assays (apoptosis; senescence; cell cycle) were performed after 3, 4 and 6 weeks of treatment. For each biological assay, we performed three biological replicates.

### Annexin V Assay

In order to perform apoptosis tests, cells were detached by trypsinization and following the washing step dissolved in 100 μL 2% FBS containing PBS. 100 μL Annexin V reagent (Millipore, Burlington, MS, USA) was added on the cells and incubated for 20 min at room temperature. Analyses were performed by using BD FACSAria III flow cytometer (Millipore, Burlington, MS, USA). For each experimental point, we analyzed at least 5,000 cells to determine the percentage of apoptotic cells.

### Cell Cycle Analysis

Detached cells were fixed with 70% ethanol at −20°C for at least 3 hours. After fixation step, cells were washed in order to remove the ethanol and stained with propidium iodide containing Cell Cycle Reagent (Millipore, Burlington, MS, USA). Analyses were performed by using Muse Cell Analyzer (Millipore, Burlington, MS, USA). For each experimental point, we analyzed at least 5,000 cells to determine the cell cycle profile.

### Senescence Associated Beta-Galactosidase Assay

Cells grown in six well plates were fixed using a 0.2% glutaraldehyde solution for 5 min at room temperature (RT). Then, the cells were washed with PBS and stained with 40 mg/ml X-gal staining solution, as reported (Debacq-Chainiaux et al., 2009). Blue-stained cells were counted from three/five different regions of each well, and the percentage of senescent cells was determined. In identifying senescent cells, we also considered other properties, such as cell size, multi-nuclei presence, and granularity. For each experimental point, we analyzed at least 1,000 cells to determine the percentage of senescent cells.

### Whole Cell Sample Preparation for Mass Spectroscopy (MS)

For every experimental point we performed two biological replicates and for each of them we did two technical replicates. Globally, we performed 32 MS analyses: 16 runs for whole cell proteome samples and 16 runs for the secretome ones. The mass spectrometry proteomics data have been deposited to the ProteomeXchange Consortium via the PRIDE (Perez-Riverol et al., 2019) partner repository with the dataset identifier PXD028349.

Samples were prepared with the InStage Tip digestion method described by Kulak and colleagues (Kulak et al., 2014). We collected 1 × 10^6^ cells each sample and, after PBS washing, cells dissolved in 100 μL Lysis Buffer (6 M Guanidinium chloride, 40 mM CAA, 10 mM TCEP, 25 mM Tris-HCl pH:8,5). Lysates were boiled for 5 min and then were sonicated in an ice-filled ultrasonic water bath for 5 min. Samples were then centrifuged at 20,000 g for 15 min, and proteins containing supernatants were collected.

We mixed 20 μL of each supernatant with 280 ng Lys-C (Promega, WI, USA) containing a 40 μL dilution buffer (25 mM Tris-HCl pH 8.5, % 10 ACN), which we put into InStage tips previously prepared by using 3 SDB-RPS extraction disks (3M Emporem, MN, USA). Mixtures were incubated overnight at 37°C. Subsequently, 1,000 ng Trypsin-Gold (Promega, WI, USA) was added to the Stage Tips, mixed well, and incubated for 4 h. Following the incubation step, a 140 μL loading buffer (1% TFA) was added to each tip and centrifuged at 2,000 g; then, the peptide-loaded disks were washed four times with a 100 μL washing buffer. Peptides were eluted from disks in three fractions, according to their hydrophobic properties, by using 60 μL of each of the three elution buffers: SDB-RPS1 (100 mM Ammonium formate, 35% ACN, 0.5% Formic Acid), SDB-RPS2 (100 mM Ammonium formate, 55% ACN, 0.5% Formic Acid), and Buffer X (80% ACN, 0.125% Ammonia). Samples were lyophilized with SpeedVac and stored at −20°C until the LC-MS/MS analysis.

### Secretome Sample Preparation for Mass Spectroscopy

MSC cultures were incubated in serum-free media for 24 h; then, 5 ml of culture medium (secretome) was collected from each culture dish without disturbing the attached cells. Culture debris was removed by centrifugation at 10,000 g for 10 min, and supernatants were used for the StartaClean beads protein pooling. Collected secretomes were incubated overnight with the beads; then, the beads were washed twice with TE Buffer (50 mM Tris 10 mM EDTA pH 7) and dried with a vacuum concentrator.

The dried beads were resuspended at 2% (w/v) in a RapiGest (Agilent, CA, USA) solution containing TEAB (Sigma, MO, USA). Then, TCEP (Sigma, MO, USA) was added to the solution at a final concentration of 20 mM. Samples were incubated at 60°C for 30 min and cooled on ice. IAA (Bio Rad, CA, USA) was added to sample solutions, and samples were incubated at RT for 15 min. Then, 200 ng Lys-C (Promega, WI, USA) was added to each sample and incubated for 4 h at 37°C. After Lys-C incubation, 800 ng Trypsin-Gold (Promega, WI, USA) was added to each sample and incubated overnight. Samples were centrifuged at 10,000 g for 1 min; peptides containing supernatants were collected and acidified with 1% TFA before being loaded into Stage Tips. These tips were prepared with C18 material: they were washed with buffer B (% 0.1 Acetic Acid, 80% ACN) and equilibrated with buffer A (% 0.1 Acetic Acid). Acidified samples were loaded onto Stage Tips, and peptide-bounded tips were washed twice with buffer A. Following the washing, buffer B was added to the tips, and samples were eluted into collecting tubes with a syringe. Samples were dried with a vacuum concentrator and stored at −20°C until LC/MS analysis.

### 5.7 LC-MS/MS Analysis

LC-MS analysis was performed with AB Sciex Triple ToF 5600+ (AB SCIEX, CA, USA) integrated with LC-MS/MS Eksigent ekspert™ nanoLC 400 System (AB SCIEX, CA, USA). Peptides were separated using nanoACQUITY UPLC 1,8 μM HSS T3 C18 column (Thermo Fisher, MS, USA) in the trap-elute mode. In order to separate the peptides, 4–40% ACN gradient was used for 240 min. Data dependent acquisition (DDA) MS/MS analysis of separated peptides was performed after electrospray ionization. Raw data analysis—generated by instrument reporting—and multiple analytical data measurements in each sample were performed with Analyst® TF v.1.6 (AB SCIEX, CA, USA). The peptides and the ion-product of the MS and MS/MS data were evaluated with PeakView (AB SCIEX, CA, USA). Generated peak-lists were evaluated in consideration of the UniProtKB-based reference library of the *Homo sapiens* species on our server with ProteinPilot 4.5 Beta (AB SCIEX, CA, USA).

### Gene Ontology, Canonical Pathways and Upstream Factors Analyses

The protein content of whole cells and secretomes was analyzed with PANTHER (http://www.pantherdb.org) and with the Ingenuity Pathway Analysis (IPA) (http://www.ingenuity.com/products/ipa).

PANTHER allowed the GO analysis by classifying protein contents according to three ontological terms: biological processes, molecular functions, and molecular classes. For PANTHER analysis, we used the statistics overrepresentation, which compares classifications of multiple clusters of lists with a reference list to statistically identify the over- or under-representation of PANTHER ontologies. Significance was set to a *p*-value of 0.05.

Differentially expressed proteins in whole cell lysates and secretome were imported into IPA to identify canonical pathways and upstream regulators. Fischer’s exact test was used to calculate a *p*-value that would determine the probability that the association between genes in the dataset and canonical pathway could be explained by chance alone. Significance was set to a *p*-value of 0.001. The IPA Upstream Regulator analysis uses known molecular interactions in the datasets to identify upstream regulators. The z-score was used to identify the significant upstream regulators.

### Statistical Analysis

Statistical significance was determined with ANOVA analysis followed by Student’s t and Bonferroni’s tests. We used mixed-model variance analysis for data with continuous outcomes. All data were analyzed with a GraphPad Prism version 5.01 statistical software package (GraphPad, CA, USA).

## Data Availability

All datasets presented in this study are included in this article.

## Supplementary Material

Supplementary Material S1 MS/MS analysis. This file details the LC-MS/MS analyses of peptides from the tryptic digestion of cell lysate samples and of secretomes. In the file the name of genes corresponding to the proteins identified in several different experimental conditions are reported.

Supplementary Material S2 Venn analysis. The file shows the proteins found in cell lysates (worksheet A) and secretomes (worksheet B) that are in common among all the experimental conditions (CTRL, 3 mM, 6 mM and 9 mM metformin treatment) and those that are only present in some. The file reports the name of the genes corresponding to the identified proteins. The Venn analysis of the GO biological process is reported in worksheet C (whole cell proteome) and D (secretome).

Supplementary Material S3 Canonical pathways identified with IPA and related Venn analysis. Panel A: This file shows the canonical pathways identified in whole cell proteomes and secretomes of control cultures and of those treated with metformin. The Venn analysis of whole proteomes and of secretomes are also reported. Panel B: the figure shows some canonical pathways identified in control culture and in those treated with metformin. The proteins present in the experimental datasets are in red.

Supplementary Material S4 Upstream transcription factors identified with IPA and related Venn analysis. This file shows the upstream transcription factors identified in the whole cell proteomes and secretomes of control cultures and of those treated with metformin. The Venn analysis of whole proteomes and of secretomes is also reported.

Supplementary Material S5 List of proteins regulated by upstream transcription factors and its related Venn analysis. This file shows the proteins regulated by some upstream transcription factors identified in whole cell proteomes and the secretomes of control cultures and those treated with metformin. The Venn analysis of whole proteomes and of secretomes is also reported.

## Abbreviations

ARD, age-related pathologies; FBS, fetal bovine serum; GO, gene ontology; LC-MS/MS, liquid chromatography-mass spectrometry/mass spectrometry; MSCs, mesenchymal stromal cells; PBS, phosphate buffer saline solution; PKR, protein kinase R; qRT-PCR, quantitative real-time PCR; ROS, reactive oxygen species; RT, room temperature; SASP, senescence associated secretory phenotype.
