# Supplementary material for: Proteomic and Biological Analysis of the Effects of Metformin Senomorphics on the Mesenchymal Stromal Cells
Source: Front Bioeng Biotechnol. 2021 Oct 5;9:730813. doi: 10.3389/fbioe.2021.730813 (PMC8524175; doi:10.3389/fbioe.2021.730813)

IPA Canonical Pathways

Metformin treated MSCs

Senescence Pathway

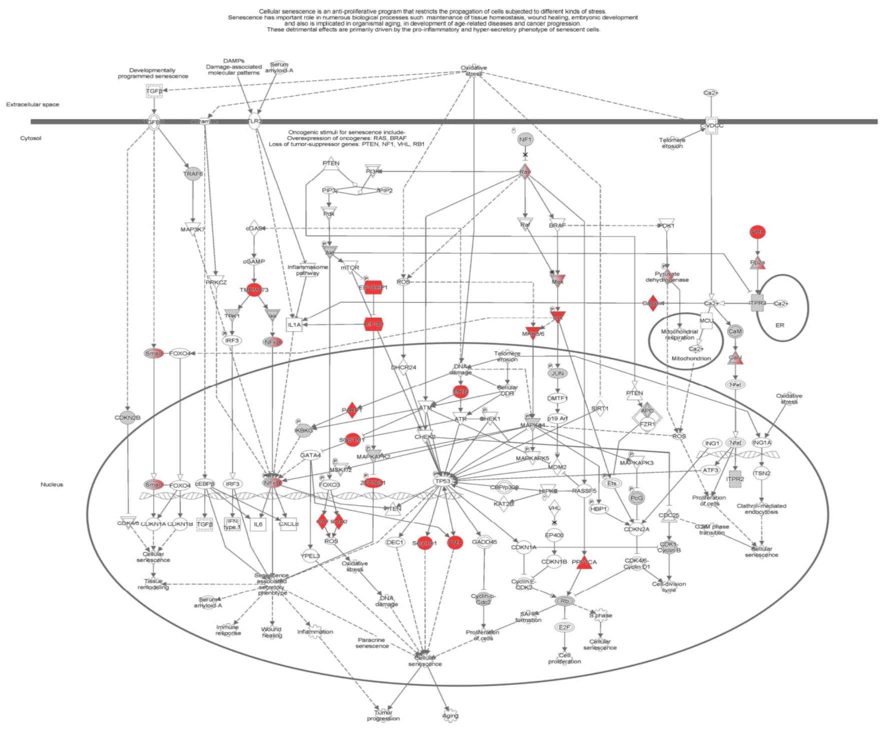

© 2000-2020 QIAGEN. All rights reserved.

control MSCs

Senescence Pathway

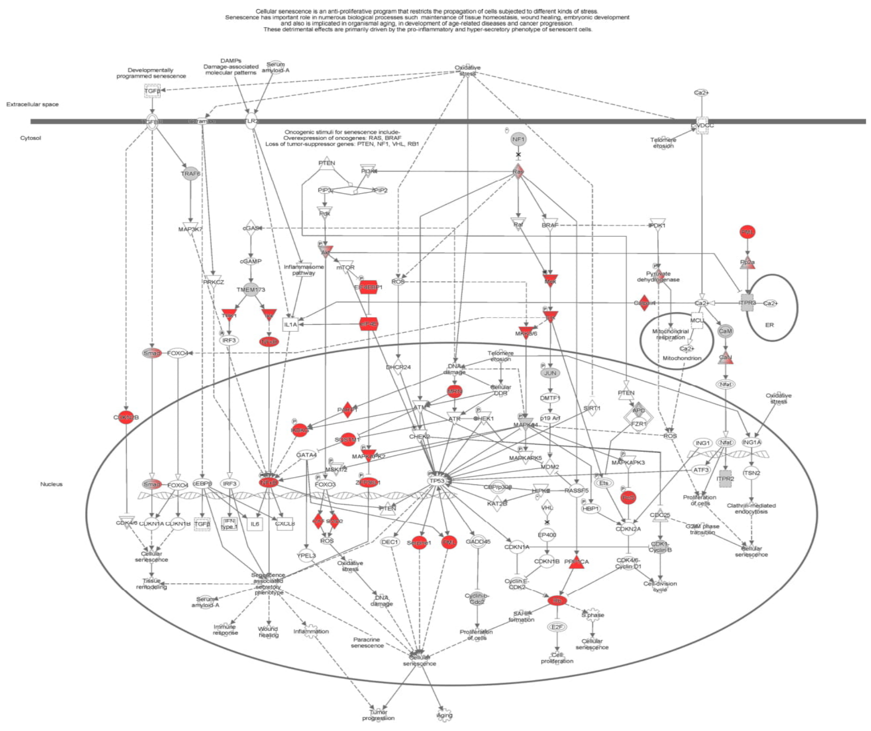

© 2000-2020 QIAGEN. All rights reserved.

Metformin treated MSCs

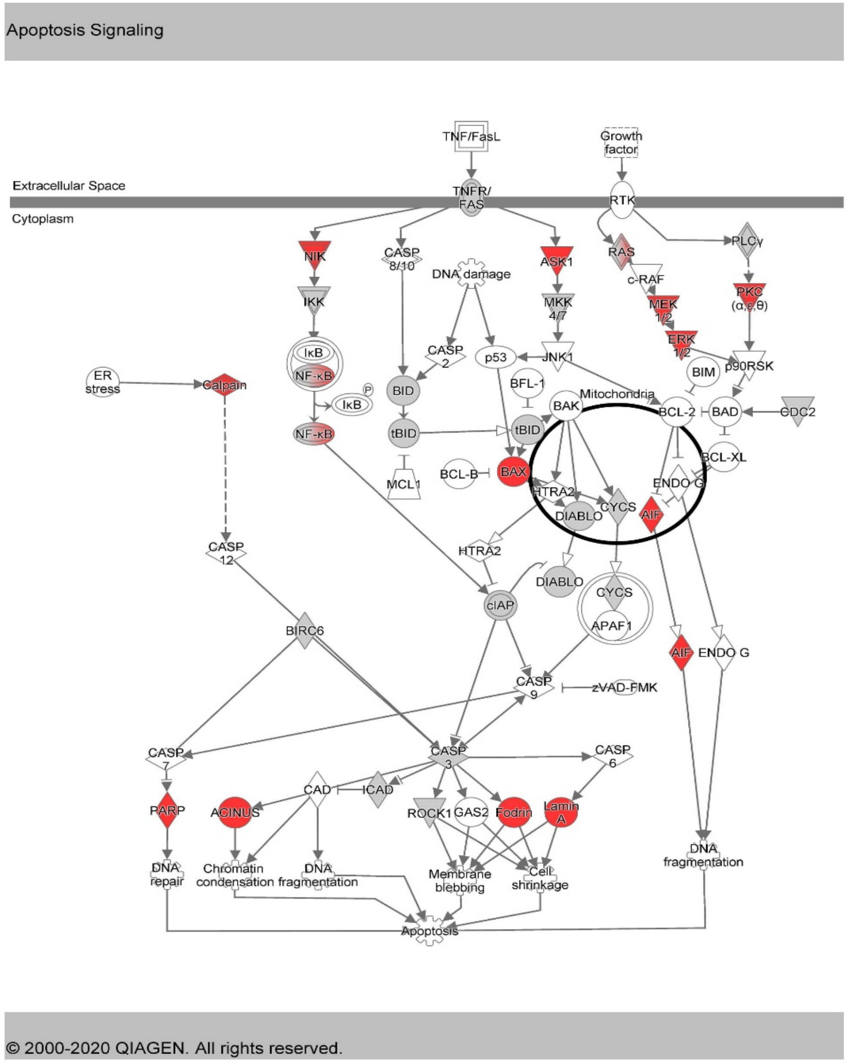

control MSCs

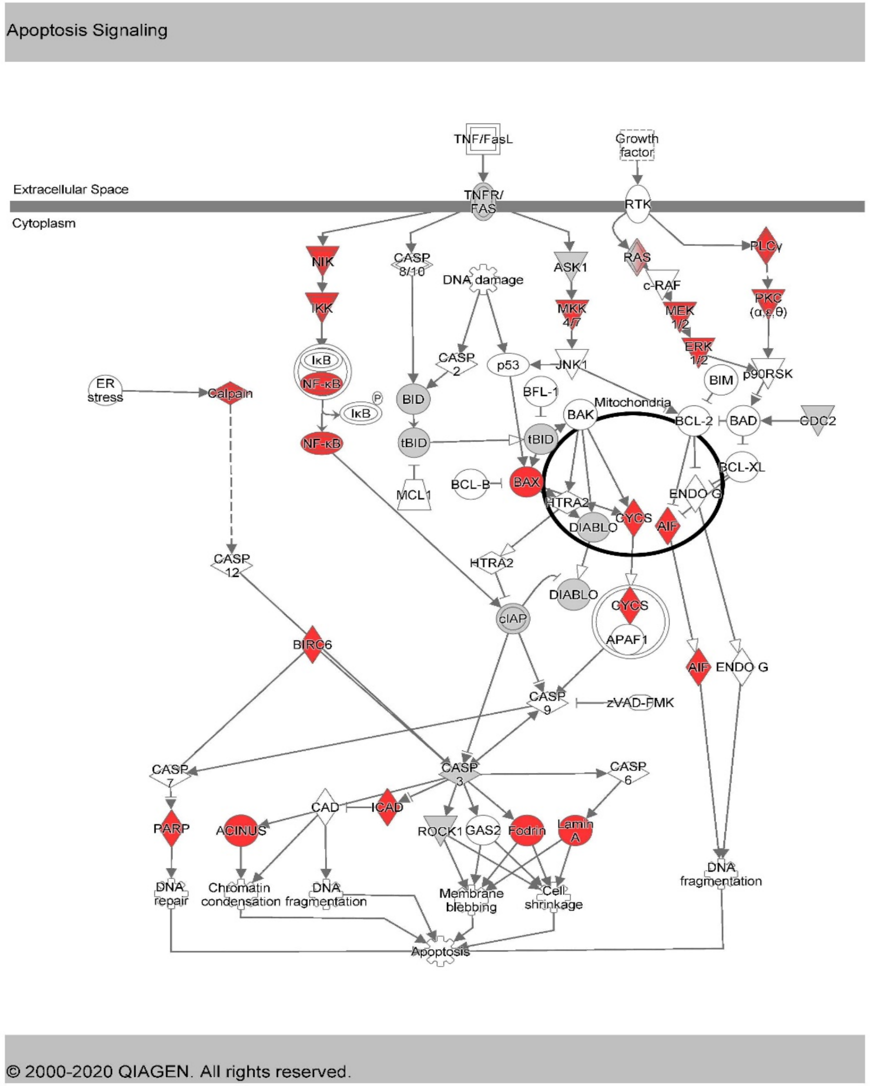

Metformin treated MSCs

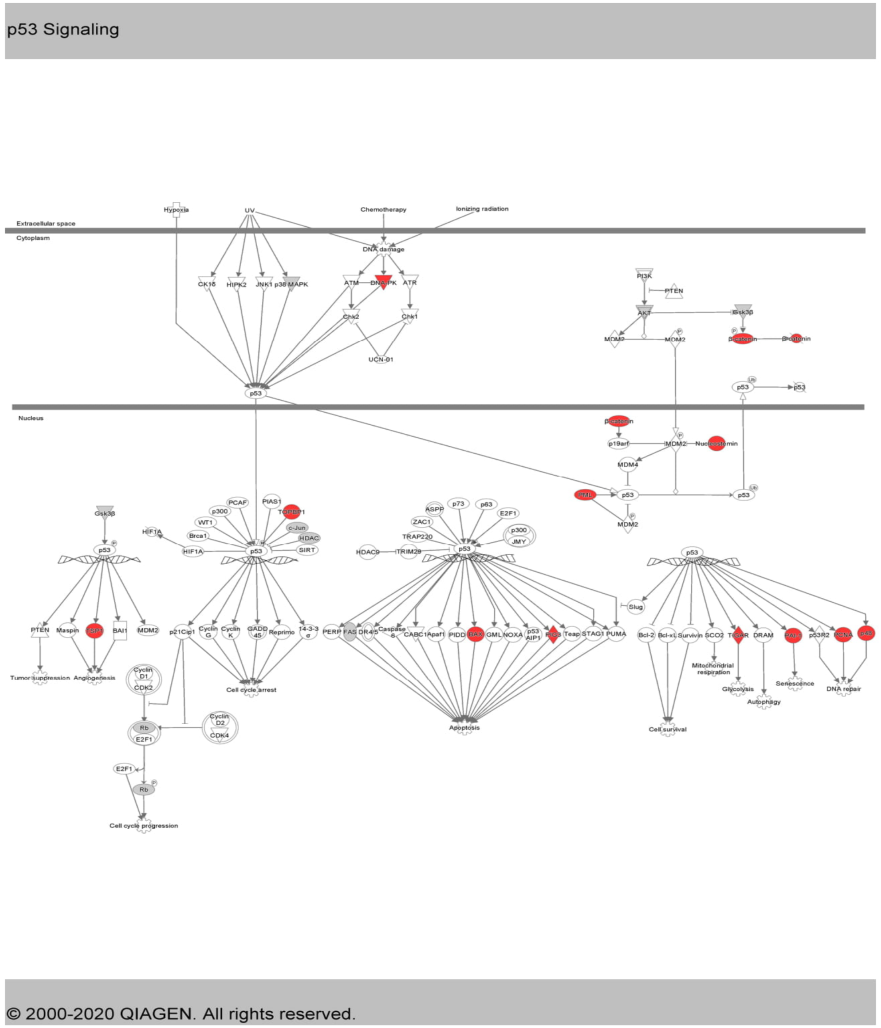

control MSCs

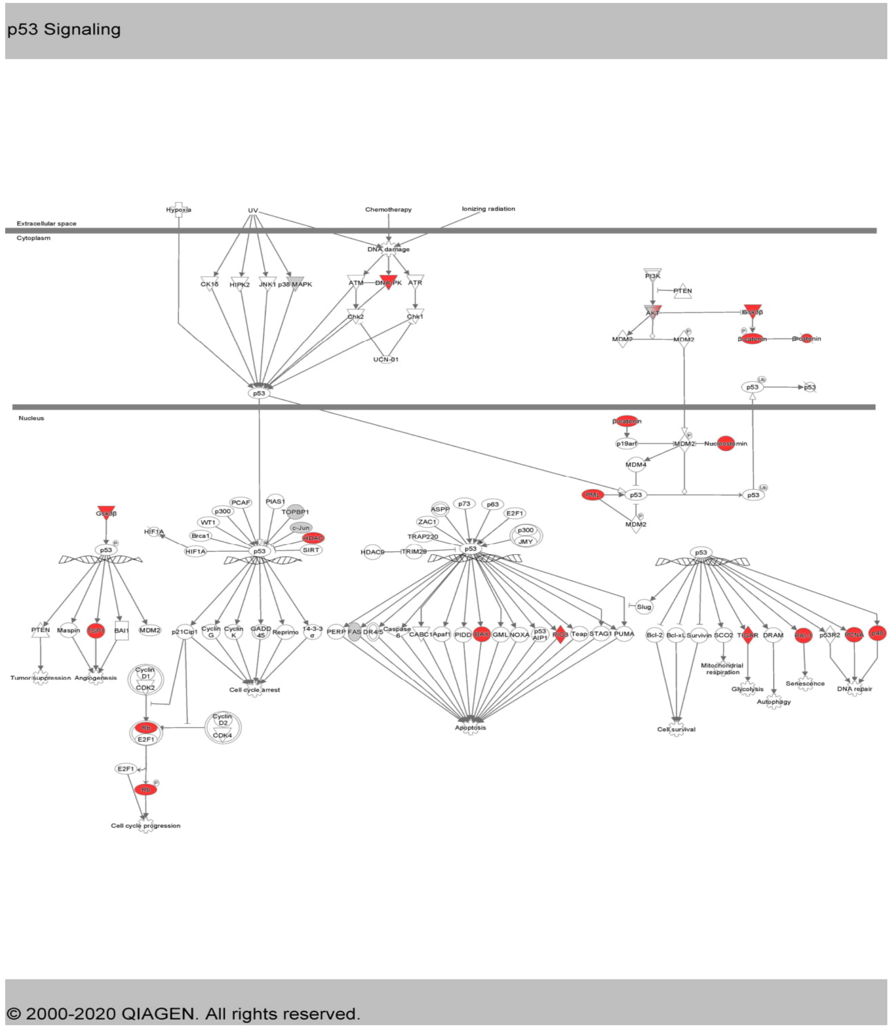

Metformin treated MSCs

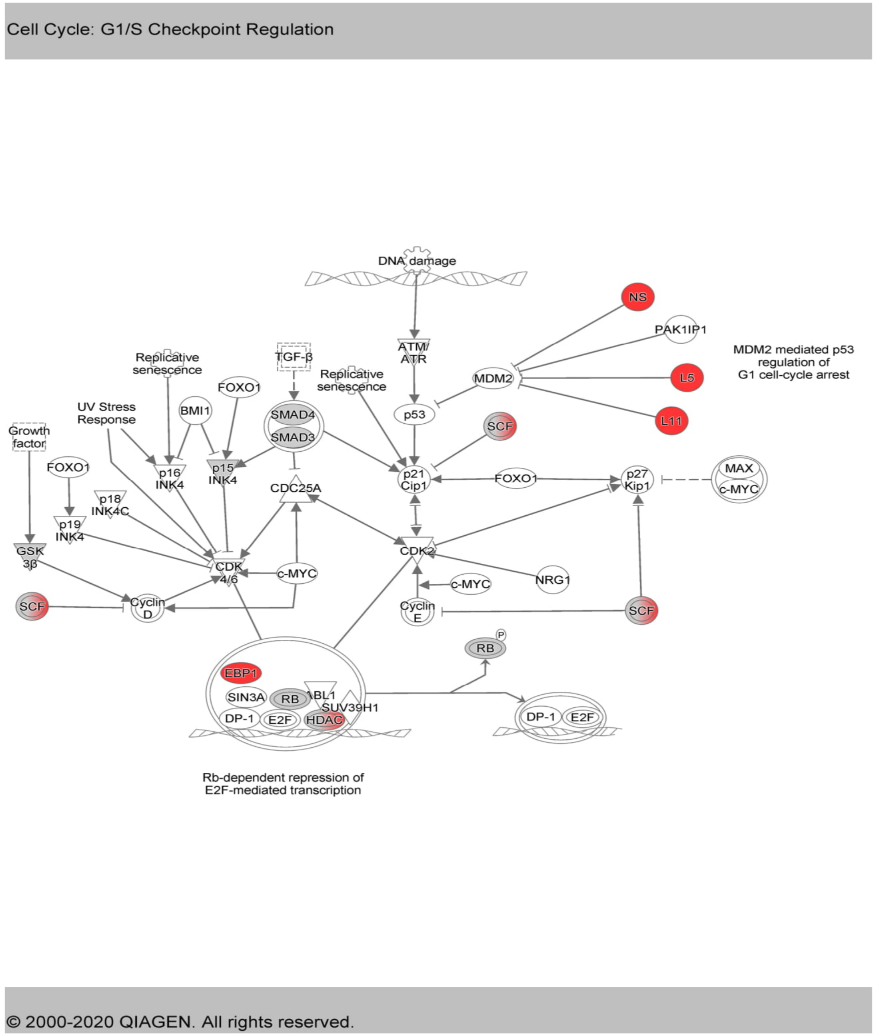

control MSCs

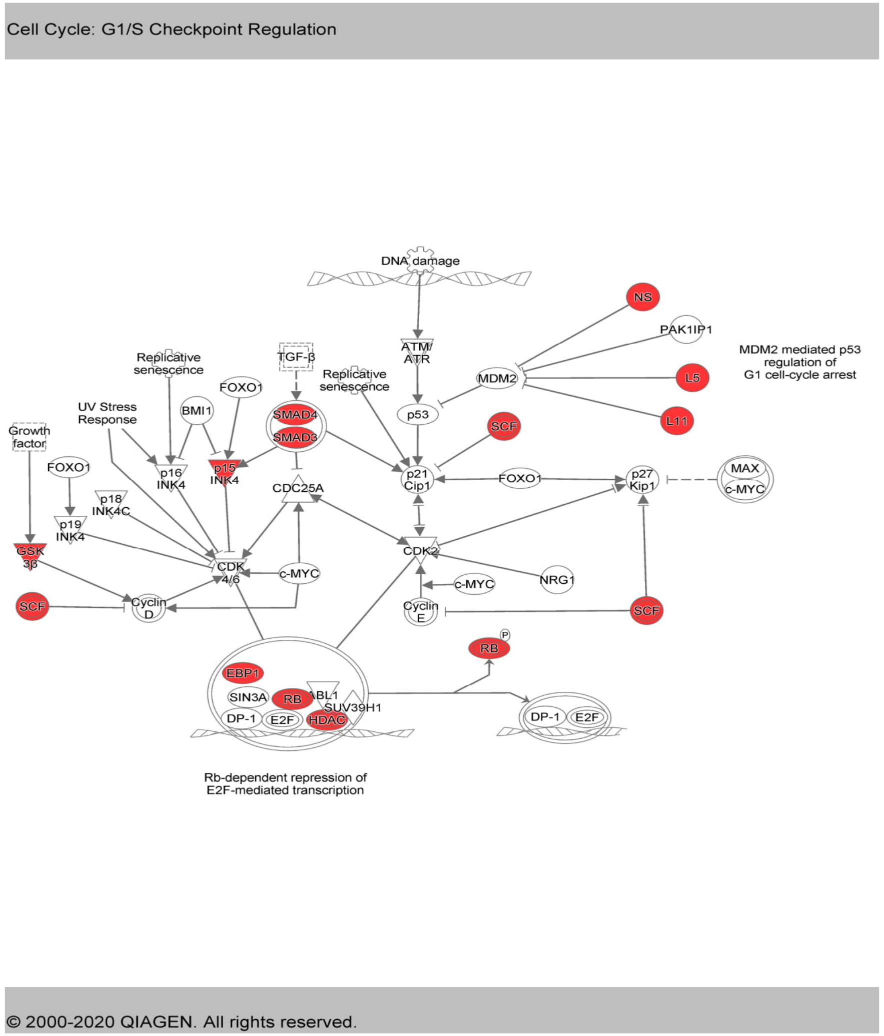

Metformin treated MSCs

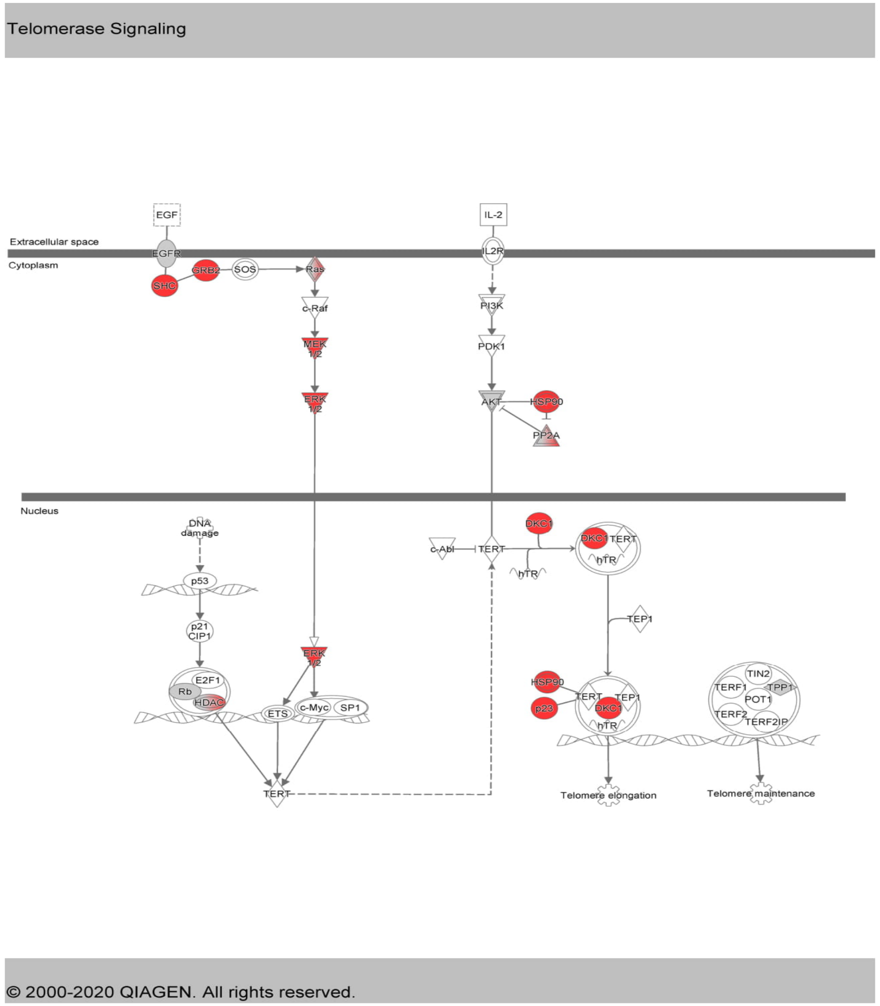

control MSCs

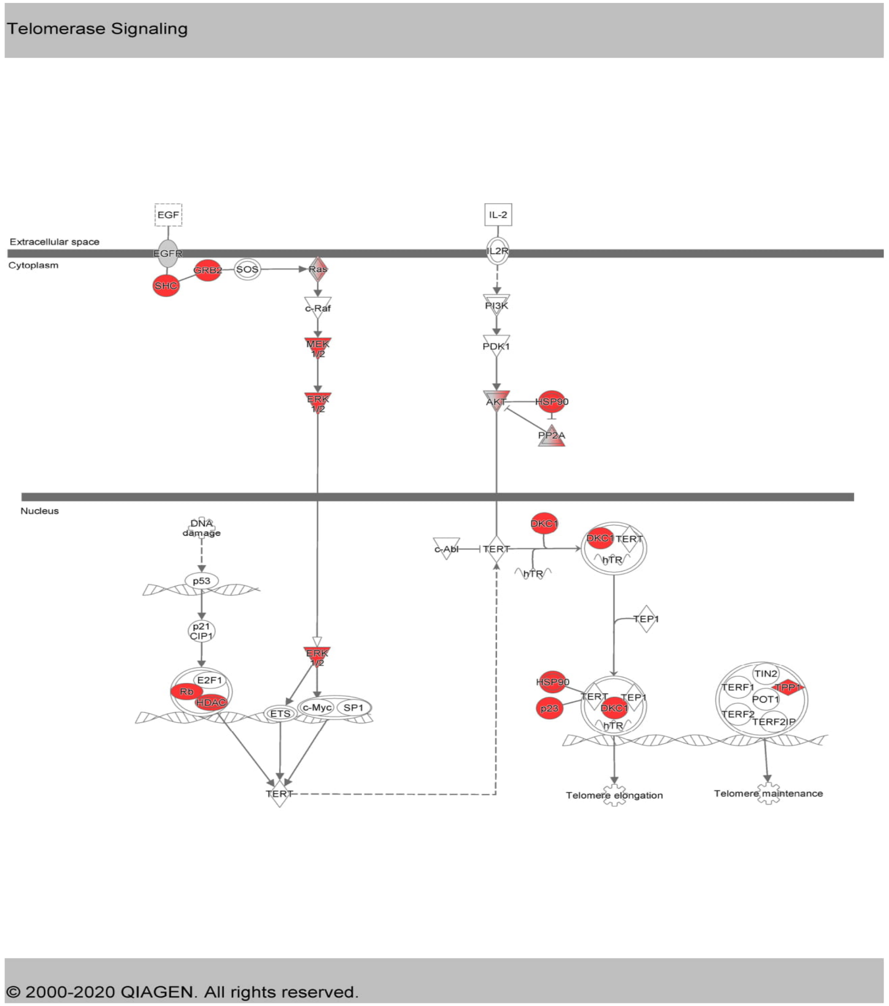

Metformin treated MSCs

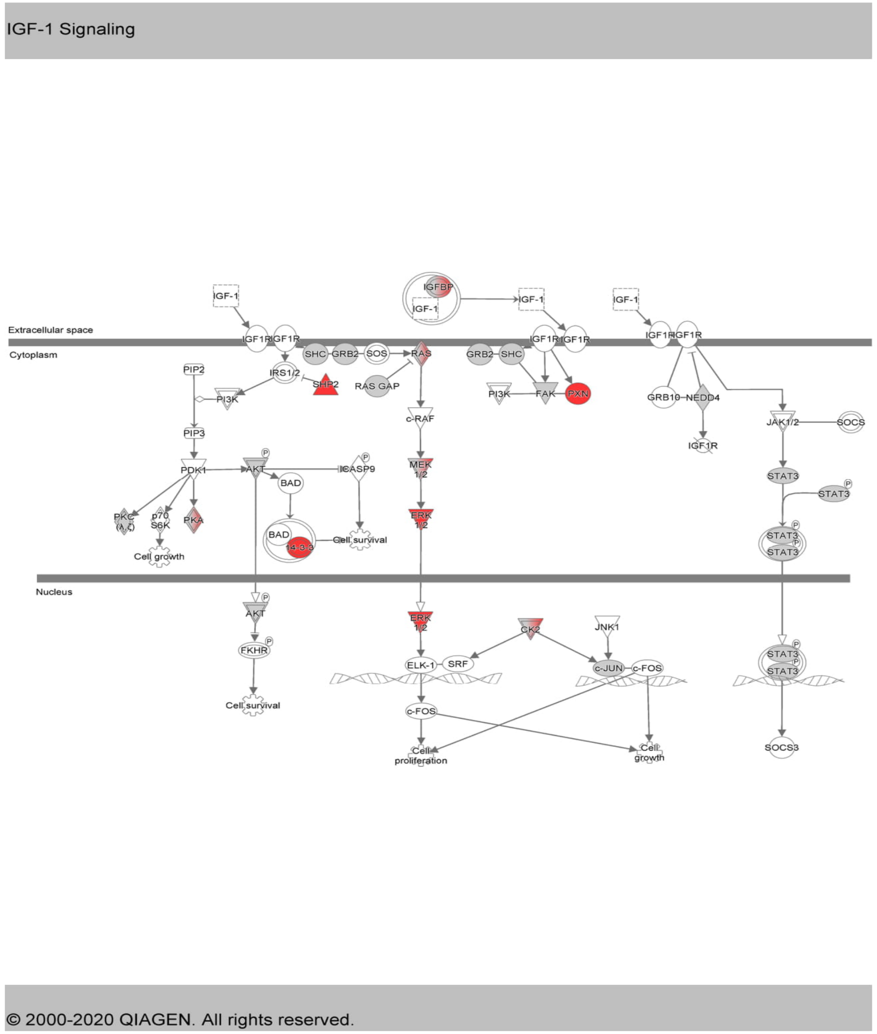

control MSCs

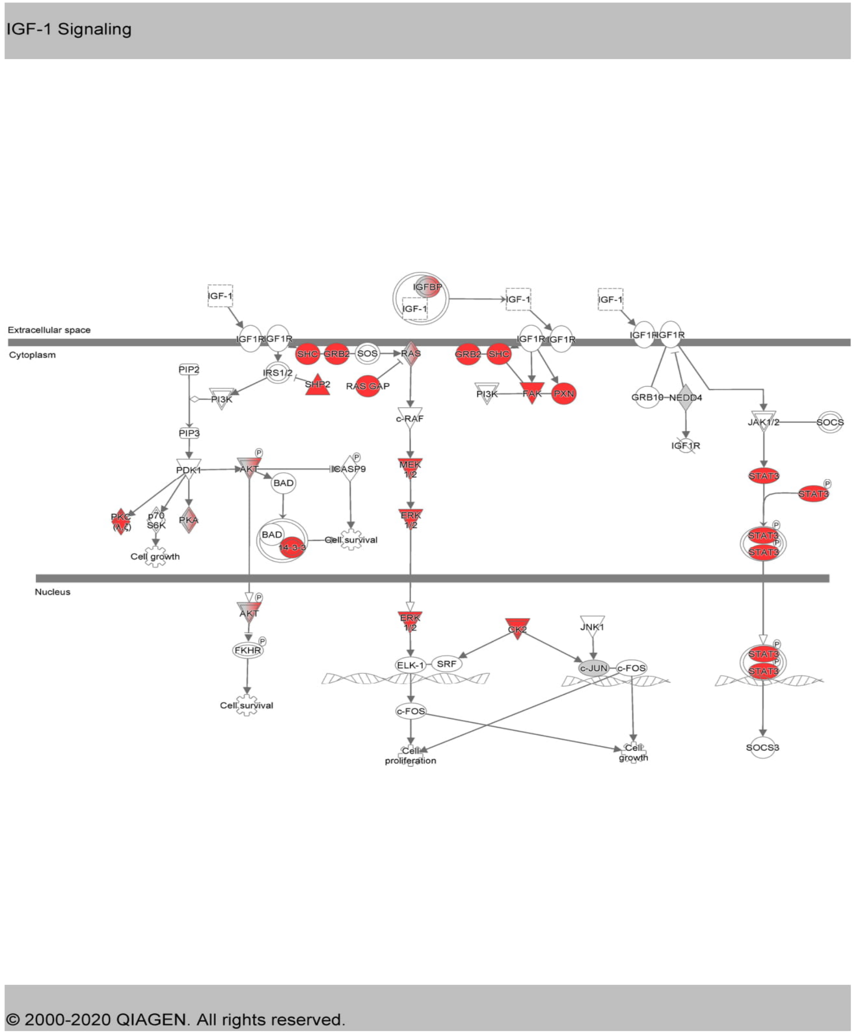

Supplement: Supplementary file 4 [file DataSheet1.PDF]
